# Severe respiratory failure caused by bacterial tracheitis in a two‐year‐old girl: ECMO and bronchoscopic management

**DOI:** 10.1111/ped.70185

**Published:** 2025-08-21

**Authors:** Kyoko Kano, Takafumi Obara, Ryo Ninomiya, Kei Honda, Naoki Yogo, Yuichiro Muto, Katsuki Hirai

**Affiliations:** ^1^ Department of Pediatrics Japanese Red Cross Kumamoto Hospital Kumamoto Japan; ^2^ Department of Emergency, Critical Care, and Disaster Medicine, Faculty of Medicine, Dentistry, and Pharmaceutical Sciences Okayama University Okayama Japan

**Keywords:** bacterial tracheitis, bronchoscopy, extracorporeal membrane oxygenation, respiratory failure

A 2‐year‐old girl (weight, 10 kg; height, 86.8 cm) presented with a barking cough 5d after contracting the influenza virus and was admitted to our pediatric intensive care unit. She was clinically diagnosed with croup and treated with nebulized epinephrine, intravenous ampicillin, and steroids. However, owing to deoxygenation and deterioration of consciousness caused by respiratory distress, she required tracheal intubation and mechanical ventilation. By Day 3, she developed septic shock and was treated with broad‐spectrum antibiotics (meropenem, vancomycin, and clindamycin), vasopressors, and renal replacement therapy. Given the atypical progression of the croup, flexible bronchoscopy (FB) was performed, revealing purulent secretions adhering circumferentially to the tracheal mucosa and extending to the peripheral bronchi (Figure 1: Day 3). Methicillin‐resistant *Staphylococcus aureus* (MRSA) was isolated from sputum cultures, confirming bacterial tracheitis.

**FIGURE 1:**
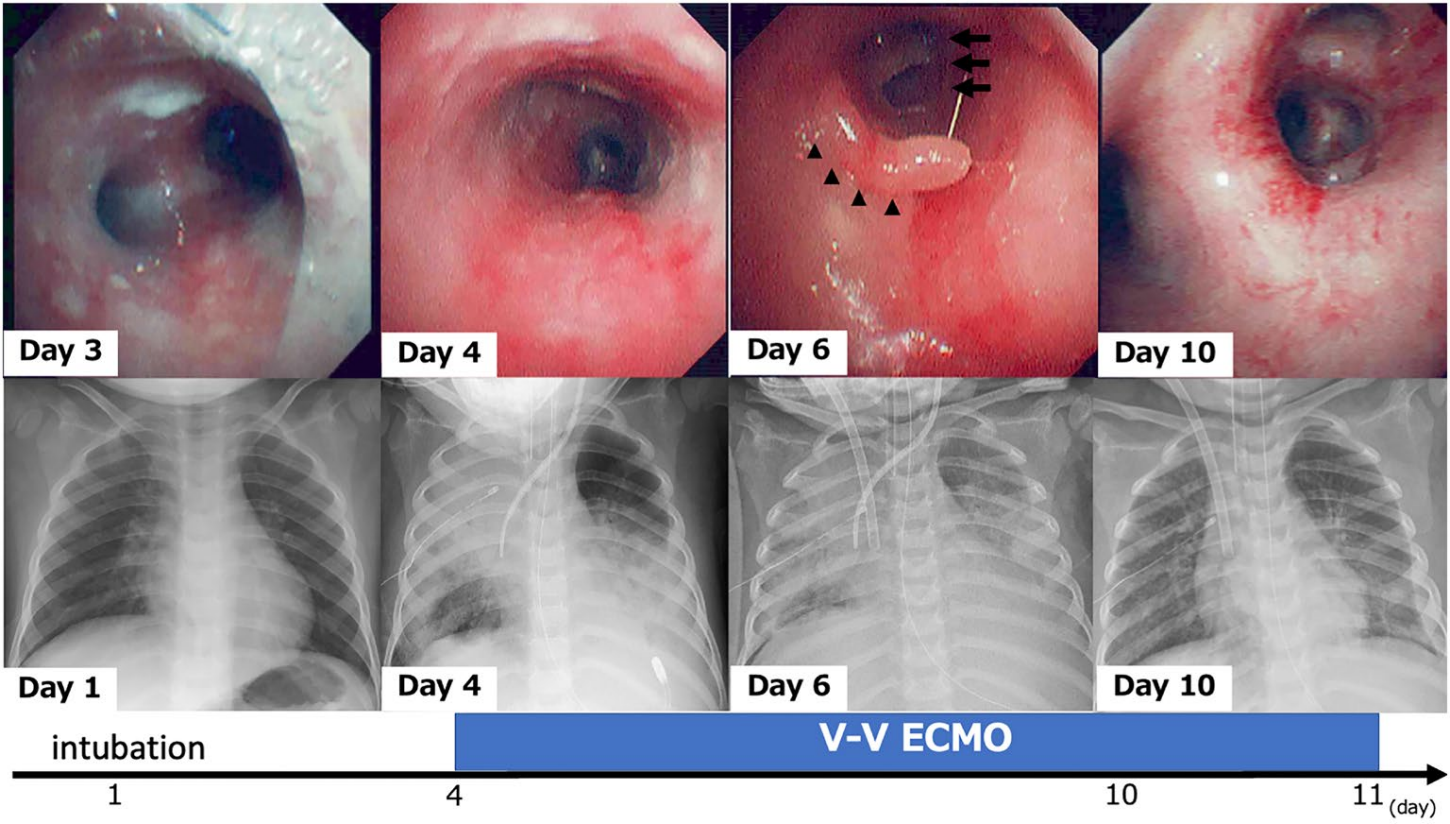
Flexible bronchoscopic images and chest X‐rays during the clinical course. Day 3: Tracheal bifurcation showing purulent secretions adhering circumferentially to the tracheal mucosa, extending to the peripheral bronchi. Day 4: Left main bronchus exhibiting continuous mucosal erythema and edematous changes. Day 6: Left main bronchus with protruding eroded tracheal mucosa (▲) and bronchial narrowing caused by obstruction from the detached tracheal mucosa (↑). Day 10: Tracheal bifurcation illustrating improvement in tracheal mucosal erythema and edema, with resolution of bronchial obstruction.

On Day 4, the ventilatory parameters had deteriorated (positive end‐expiratory pressure [PEEP], 8 cm H_2_O; fraction of inspired oxygen [FiO_2_], 1.0; pressure control, 38 cm H_2_O; and respiratory rate [RR], 26). Despite right pleural drainage and nitric oxide inhalation, she developed hypoxemia (oxygenation index, 33) and respiratory acidosis. Veno‐venous extracorporeal membrane oxygenation (V‐V ECMO) was initiated via right cervical and femoral veins. Initial V‐V‐ECMO settings include a Biocube oxygenator (NIPRO, Japan); right internal jugular vein using BioMedicus™NextGen catheter 14Fr (Medtronic, Mexico); right femoral vein using BioMedicus catheter 14Fr (Medtronic, Mexico); blood flow, 1.0 L/min, speed, 2900 rpm, oxygen sweep gas, 2.0 L/min; and the fraction of oxygen 1.0 using heparin. FB revealed severe mucosal erythema, tracheobronchial detachment, and peripheral airway obstruction (Figure 1: Day 4). Following ECMO initiation, her condition stabilized, allowing for daily evaluation via FB. On Day 6 (ECMO Day 3), the bronchi showed persistent inflammation, edema, and mucosal erosion causing airway narrowing (Figure 1: Day 6). By Day 10 (ECMO Day 7), mucosal healing was evident, and airways had reopened after clearing secretions and debris (Figure 1: Day 10). On Day 11, V–V ECMO was discontinued and she was transitioned to mechanical ventilation (PEEP 10 cm H_2_O; FiO_2_, 0.6; pressure control, 25 cm H_2_O; and RR 20). She was extubated on Day 18, and completed a 3‐week anti‐MRSA course. The patient was discharged on Day 42 with no physical and neurological deficits.

Bacterial tracheitis is a rare pediatric condition, with an estimated annual incidence of 0.1 cases per 100,000 children.^1^ Diagnosis is based on upper airway obstruction and bronchoscopic findings of purulent secretions adhering to the tracheal mucosa.^2^ Approximately 70–90% of patients require intubation; however, following initial intubation to relieve airway obstruction, patients often show dramatic improvement.^1^ Comprehensive treatment, including mechanical ventilation and broad‐spectrum antibiotics targeting MRSA, contributes to a generally favorable prognosis. Nonetheless, severe complications, including pneumothorax (1.3%) and acute respiratory distress syndrome (ARDS) (1.0%), have been reported, and conventional ventilation often fails to improve oxygenation.^1^

For acute and reversible respiratory failure, V–V ECMO provides essential support for breathing and oxygen delivery, helping the patient recover until the infection resolves or lung function improves.^3^ However, few cases of V–V ECMO for bacterial tracheitis have been reported, possibly because respiratory function often improves rapidly with conventional management.^4^ In this case, the patient experienced transient circulatory instability from septic shock and severe respiratory failure characterized by tracheal mucosal edema and extensive obstruction resulting from the extension of the detached mucosa to the peripheral bronchi. Despite the stabilization of the patient's hemodynamic status, challenges with conventional mechanical ventilation and the patient's small body size, which limits effective suctioning of the obstruction, prompted the decision to initiate V–V ECMO.

Generally, most patients with bacterial tracheitis improve with relatively short‐term intubation of 2–4 days. However, management may be prolonged if the lung parenchyma becomes involved or ARDS develops.^5^ Even in this case, earlier bronchoscopy during intubation might have allowed for prompt diagnosis and timely escalation to broad‐spectrum antibiotics, potentially preventing progression to severe disease. The sloughed mucosa remains fragile, and bronchoscopic suctioning and removal pose persistent challenges, even during ECMO. Nevertheless, this case highlights the successful application of prolonged management, with daily bronchoscopic assessment safely conducted under V–V ECMO support.

In addition, serial bronchoscopic evaluations offered real‐time, direct visualization of tracheobronchial recovery, including resolving mucosal erythema and reopening of peripheral bronchi. These findings, which paralleled improvements in ventilatory parameters and radiographic appearance, provided an objective basis for assessing disease progression and confidently determining the timing of ECMO weaning. In our case, bronchoscopy served as a valuable adjunct to standard clinical indicators, enhancing decision‐making for decannulation. Given its safety under ECMO support, bronchoscopy might be a useful tool for assessing disease severity and guiding weaning decisions in similar cases of bacterial tracheitis.

In conclusion, we successfully managed a severe case of respiratory failure caused by pediatric bacterial tracheitis using V–V ECMO and daily FB to monitor disease progression. For life‐threatening respiratory failure, prompt administration of ECMO therapy offers an effective, lifesaving option, enabling full recovery.

## AUTHOR CONTRIBUTIONS

K.K. and T.O. wrote the manuscript. R.N, K.H, N.Y, Y.M, and K.H. supervised and critically revised the manuscript. All authors met the criteria for authorship contribution based on the recommendations of the International Committee of Medical Journal Editors (ICMJE). All authors read and approved the final manuscript.

## CONFLICT OF INTEREST STATEMENT

The authors declare no conflicts of interest.

## CONSENT FOR PUBLICATION

Written approval was obtained from the patient's parents.

## Data Availability

Data sharing not applicable to this article as no datasets were generated or analysed during the current study.
